# A Predictive Model for Nonsentinel Node Status after Sentinel Lymph Node Biopsy in Sentinel Lymph Node-Positive Chinese Women with Early Breast Cancer

**DOI:** 10.1155/2022/7704686

**Published:** 2022-02-24

**Authors:** Lifang He, Peide Liang, Huancheng Zeng, Guangsheng Huang, Jundong Wu, Yiwen Zhang, Yukun Cui, Wenhe Huang

**Affiliations:** ^1^Breast Center, Cancer Hospital of Shantou University Medical College, Shantou 515000, Guangdong Province, China; ^2^Guangdong Provincial Key Laboratory for Breast Cancer Diagnosis and Treatment, Cancer Hospital of Shantou University Medical College, Shantou 515000, Guangdong Province, China; ^3^Department of Thyroid and Breast Surgery, Dongguan Houjie Hospital, Dongguan 523000, Guangdong Province, China; ^4^Department of Breast and Thyroid Surgery, Xiang'an Hospital of Xiamen University, No. 2000, Xiang'an East Road, Xiamen 361101, Fujian Province, China; ^5^Key Laboratory for Endocrine-Related Cancer Precision Medicine of Xiamen, Xiamen 361101, Fujian Province, China

## Abstract

**Background:**

Axial lymph node dissection (ALND) is needed in patients with positive sentinel lymph node (SLN). ALND is easy to cause upper limb edema. Therefore, accurate prediction of nonsentinel lymph nodes (non-SLN) which may not need ALND can avoid excessive dissection and reduce complications. We constructed a new prognostic model to predict the non-SLN metastasis of Chinese breast cancer patients.

**Methods:**

We enrolled 736 patients who underwent sentinel lymph node biopsy (SLNB); 228 (30.98%) were diagnosed with SLNB metastasis which was determined by intraoperative pathological detection and further accepted ALND. We constructed a prediction model by univariate analysis, multivariate analysis, “R” language, and binary logistic regression in the abovementioned 228 patients and verified this prediction model in 60 patients.

**Results:**

Based on univariate analysis using *α* = 0.05 as the significance level for type I error, we found that age (*P*=0.045), tumor size (*P*=0.006), multifocality (*P*=0.011), lymphovascular invasion (*P*=0.003), positive SLN number (*P*=0.009), and negative SLN number (*P*=0.034) were statistically significant. Age was excluded in multivariate analysis, and we constructed a predictive equation to assess the risk of non-SLN metastasis: Logit(*P*)=Ln(*P*/1 − *P*)=0.267*∗a*+1.443*∗b*+1.078*∗c*+0.471*∗d* − 0.618*∗e* − 2.541 (where “*a*” represents tumor size, “*b*” represents multifocality, “*c*” represents lymphovascular invasion, “*d*” represents the number of metastasis of SLN, and “*e*” represents the number of SLNs without metastasis). AUCs for the training group and validation group were 0.715 and 0.744, respectively. When setting the risk value below 22.3%, as per the prediction equation's low-risk interval, our model predicted that about 4% of patients could avoid ALND.

**Conclusions:**

This study established a model which demonstrated good prognostic performance in assessing the risk of non-SLN metastasis in Chinese patients with positive SLNs.

## 1. Introduction

In the 2021 report, the International Agency for Research on Cancer (IARC) showed there were 2.26 million new breast cancer cases in 2020 worldwide, comprising the world's largest cancer incidence, with a mortality rate that remains the second leading cause of death for women with cancer. China's annual incidence of new breast cancer cases is 420,000, accounting for 18.6% of the world's total new breast cancer cases, and it is also the largest number of new cancer cases for women in China [1].

Early-stage breast cancer is mainly treated with surgery, chemotherapy, endocrine therapy, radiotherapy, or targeted therapy [2, 3]. Axillary lymph node dissection (ALND) has been a standard method in breast cancer surgery, since the inception of Halsted radical mastectomy, and has always been the gold standard for assessing axillary lymph node metastasis and determining cancer stage. However, its complications, such as upper limb dysfunction, upper arm lymphedema, and sensory disorders, seriously reduce the patients' quality of life [4]. With the development of the “precision medicine” surgical treatment approach [5], sentinel lymph node biopsy (SLNB), first introduced for breast cancer surgery by Krag et al. [6, 7], has gradually replaced axillary surgery for early breast cancer treatment. It is an accurate method for assessing axillary lymph node status that could avoid severe complications and improve postoperative quality of life. However, only about 35% of all breast cancer patients who undergo SLNB have SLN metastases [8]. A retrospective study also showed that about 40%–70% of patients who had a positive SLN and accepted routine ALND had no tumor metastasis in their non-SLNs [9]. Therefore, the necessity of ALND after SLNB is still debated.

Preoperative prediction of SLN and non-SLN status has gained growing attention among doctors and scientists. Identifying patients who may not need ALND after SLNB requires an accurate method to predict non-SLN metastasis. Van et al. adopted the Memorial Sloan Kettering Cancer Center (MSKCC) model to predict the likelihood of non-SLN metastasis. This model included nine clinical or pathological variables and had an area under the curve (AUC) of 0.77 in a subsequent prospective study on 373 patients [10]. Many clinical centers have verified this model, but most found AUCs between 0.58 and 0.72 due to differences between countries and populations [10–12]. Most of the current non-SLN metastasis prediction models are based on patients from Western countries [13, 14]. However, the numerous Chinese breast cancer patients differ from Western patients in race, diet, culture, and medical standards. Therefore, developing models suitable for Chinese populations is a necessity.

## 2. Materials and Methods

### 2.1. Case Collection

From November 2009 to December 2018, female patients (*n* = 736) who received SLNB were diagnosed as having primary breast cancer by preoperative or intraoperative biopsy in the Breast Cancer Center, Affiliated Cancer Hospital of Shantou University Medical College (Shantou, Guangdong Province, China). We recruited 228 of them for a retrospective training group. These cases fulfilled the following criteria: (1) primary invasive breast cancer was detected by preoperative needle biopsy or intraoperative freezing pathology, (2) patients met the cT1-3N0M0 stage according to the eighth edition of the American Joint Committee on Cancer (AJCC) staging manual, (3) patients had not received prior neoadjuvant therapy, (4) patients had undergone SLNB and tumor metastasis was observed in the SLNs, including isolated tumor cells, tumor micrometastases, or macrometastases, (5) an experienced surgical team performed the SLNB, (6) patients accepted further ALND, and (7) patients had no prior history of cancer. We continued to recruit 60 patients who met the inclusion criteria in our hospital's breast center from January 2019 to July 2020 for preliminary verification of the model. The Ethics Committee of the Cancer Hospital of Shantou University Medical College approved this study (No. 2021102).

### 2.2. Surgery and Pathology

SLNB was performed using 2 ml subcutaneously injected blue dye injection (Jichuan Pharmaceutical Group Co., Ltd., 10 mg/mL, Jiangsu) and 2 ml subcutaneously injected indocyanine green injection (Dandong Medical Innovation Pharmaceutical Co., Ltd., 12.5 mg/ml, Liaoning). The staining tracer was injected into the areolar area, tumor surface, or subcutaneous tissue adjacent to the tumor, and SLNB was performed 5–10 minutes later. During SLNB, the main procedure consisted of looking for lymphatic vessels with blue staining or infrared irradiation in the axilla and then exploring the SLNs along the lymphatic vessels. We regarded stiff and swollen nodes near the blue-stained lymphatic vessels as the SLNs. A professional pathologist immediately examined all SLNs and communicated the result to the surgical team for a second judgment. In cases with tumor metastasis in the SLNs, we routinely dissected the level I or II axillary lymph nodes. If lymph nodes in level II displayed metastases, we also dissected axillary lymph nodes in level III [15]. After the operation, all specimens were paraffin-embedded for immunohistochemistry. We evaluated the pathological stage and molecular subtype of the tumor according to the St. Gallen International Expert Consensus on the Primary Therapy of Early Breast Cancer 2013 and College of American Pathologists clinical practice guideline [16, 17].

### 2.3. Data and Analysis

**Figure 1:**
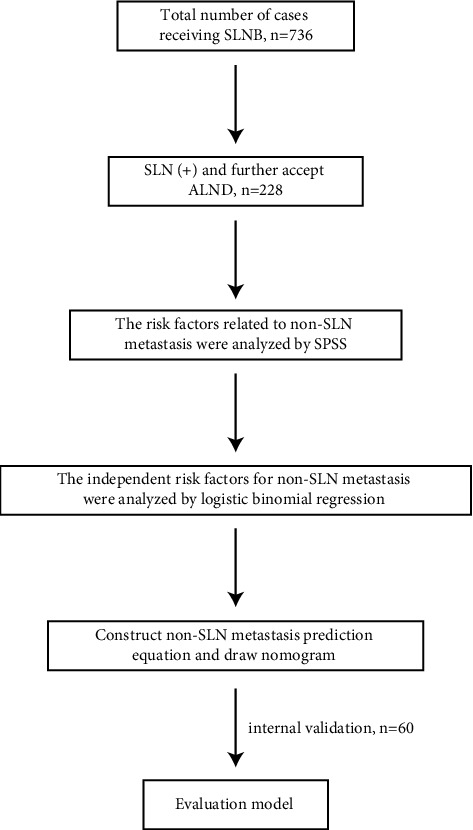
Project flow chart.

The flowchart of variable screening, nomogram model construction, and model evaluation is shown in Figure 1. We analyzed the data using SPSS 19.0 and performed a normality test before calculating the median, mean, and standard deviation. Enumeration data are expressed as frequencies or composition ratios. Ranked data are expressed as frequencies. We compared the data using a t-test for two independent samples or nonparametric tests. Enumeration data were analyzed using the chi-square test or Fisher's exact test. Ranked data were analyzed using the rank-sum test. The independent risk factors for non-SLN tumor metastasis were analyzed by logistic binomial regression. The first error level was set as *α* = 0.05 in all of the tests above, and *P* < 0.05 was considered to indicate statistical significance.

## 3. Results

### 3.1. Patient Characteristics

In the training group, 228 female patients with early breast cancer had at least one positive SLN and underwent complete ALND. Among them, 112 were positive for non-SLN metastases after ALND, indicating that the non-SLN metastasis incidence was 49.1%. The average tumor size was 3.31 ± 1.29 cm, and the average age was 51 years (28–86 years). We dissected 3,636 lymph nodes from patients who received ALND, with an average of 13.05 ± 5.3 per patient. The total dissected number of SLNs was 661, and the average number of total SLNs, positive SLNs, and negative SLNs was 2.90 ± 1.48, 1.43 ± 0.73, and 1.46 ± 1.35, respectively. The validation and training groups had similar data distributions (Table 1).

### 3.2. Correlation Analysis for Non-SLN Metastasis

According to the univariate analysis, the variables significantly associated with metastasis in the non-SLNs include age, clinical tumor size, multifocality, number of positive SLNs, number of negative SLNs, and lymphovascular invasion (*P* < 0.05) (Table 2). Multivariate analysis confirmed that clinical tumor size, multifocality, lymphovascular invasion, number of positive SLNs, and number of negative SLNs were independent predictors of non-SLN metastasis (Table 3).

### 3.3. Establishment of a Predictive Model for Non-SLN Metastasis

**Figure 2:**
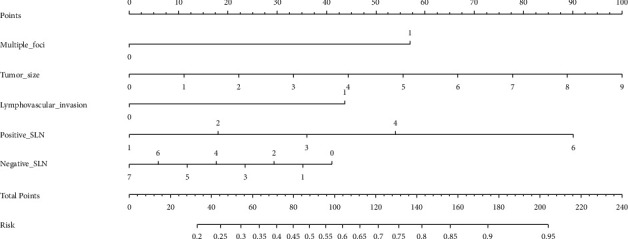
Nomogram for predicting the likelihood of non-sentinel lymph node (non-SLN) metastases in SLN-positive breast cancer patients. Substitute the variables according to the actual situation in rows 2 to 6. The relative score for each variable is obtained by drawing a vertical line between each variable and the first row (Points). Then, the total points should be calculated and placed in row 7. Finally, find the predicted P, indicating the probability of non-SLN metastasis, in row 8.

From the results of binary logistic analysis, we established the following predictive equation: Logit(*P*)=ln(*P*/1 − *P*)=0.267 × *a*+1.443 × *b*+1.078 × *c*+0.471 × *d* − 0.618 × *e* − 2.541. During the calculation, we substituted the measured values of clinical tumor size, positive SLN number, and negative SLN number into the formula. We assigned a value of 0 or 1 for both multifocality and lymphovascular invasion, depending on the actual situation (Table 4). Using the binary logistic regression analysis results, using the “R” language (version 3.5.3), we constructed a nomogram providing the probability of non-SLN metastasis (Figure 2).

### 3.4. Validation and Application of the Predictive Model

**Figure 3:**
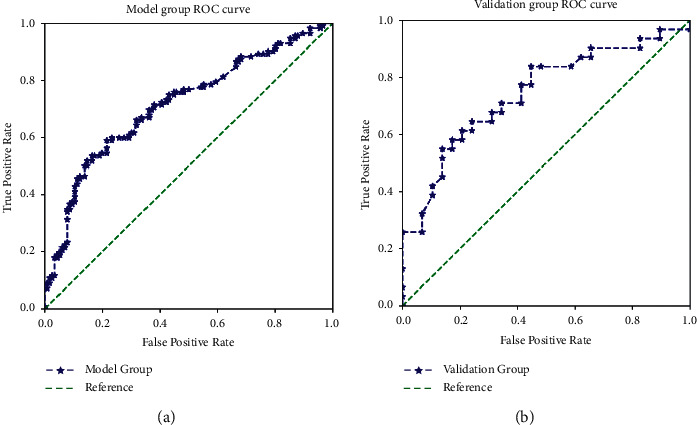
Receiver operating characteristic (ROC) curve, for the predictive equation, in the training group ((a) the area under the ROC curve was 0.715, *n* = 228) and validation group ((b) the area under the ROC curve was 0.744, *n* = 60).

The AUC was 0.715 in the training group and 0.744 in the validation group, indicating consistent prediction performance in both groups (Figures 3(a) and 3(b)). Using the model in the clinic (to avoid ALND for low-risk SLN metastasis patients) requires setting a low-risk cutoff value in the model. When accepting a low-risk cutoff value of ≤14.2%, about 2% of patients could be safely exempted from ALND, and the sensitivity was 100% in the training group. When accepting a low-risk cutoff value of ≤22.3%, the sensitivity for both groups was above 90% and the false-negative rate was below 10% (Tables 5 and 6). When accepting a low-risk cutoff value of ≤31.2% in the training group, the model correctly predicted that about 13% of patients had no non-SLN metastasis and the false-negative rate was below 10%.

## 4. Discussion

With low trauma, high sensitivity, and high accuracy, SLNB has gradually replaced ALND as the preferred method of axillary treatment for most ALN-negative patients with early breast cancer [18]. Studies have pointed out that not all SLN-positive patients have non-SLN metastases. Only about 30%–50% of patients positive for SLN metastases have non-SLN metastases [19]. In our study, 112 patients (49.1%) displayed non-SLN metastases, consistent with the literature [19]. Clinical trials such as IBCSG 23 [20] and Z0011 [21] have questioned the necessity of ALND for positive SLN patients and suggest that SLNB (alone or in combination with standard postoperative treatment) can achieve good local control without subsequent ALND for some SLN-positive low-risk populations.

The MSKCC nomogram is the most well-known multivariable model and has been used and verified in many hospitals, especially in Western countries. However, the AUC values obtained with the MSKCC nomogram fluctuate wildly depending on countries and populations. Wu et al. [13] used Chinese breast cancer patients to test the prognostic value of six standard models and obtained AUC values measured by the SNUH (Seoul National University Hospital), Louisville, MSKCC, Tenon, Stanford, and SCH (Shanghai Cancer Hospital) models of 0.706, 0.702, 0.677, 0.673, 0.432, and 0.674, respectively. Thus, models have different predictive abilities, with AUC values ranging from 0.6 to 0.8. Each model was constructed from clinical and pathological data for specific populations and therefore has the highest predictive value for that specific population but may not apply to other subjects. The SCH model is the first predictive non-SLN model in China and includes tumor size, number of negative SLNs, number of positive SLNs, vascular infiltration, and SLN tumor metastasis as variables. Although it achieved an AUC of 0.79 for its studied population, the results obtained for patients from other Chinese regions were not satisfactory (AUC = 0.674) [13, 22]. Our model achieved AUCs higher than 0.7 for both the training and validation groups. However, our validation group only contained 60 cases. Therefore, our model requires validation in other large independent populations before becoming feasible for clinical use. In the prediction model, each factor does not play a decisive role and each factor may affect lymph node metastasis. The clinical and pathological factors that may affect non-SLN metastasis are complex, with the most commonly associated risk factors being age, clinically positive lymph nodes, tumor size, tumor location, multifocality, pathologic type, neurovascular invasion, histological grade, number of positive and negative SLNs, positive SLN ratio, size of the SLN metastases detected, hormone receptor status, SLN micrometastases, and extracapsular invasion in the SLN [10, 23–26]. Among them, tumor size, neurovascular invasion, and positive SLN number have been common strong independent factors in multiple tests.

Our model involves only five independent factors, including the three essential factors mentioned above. It is simpler than previous models and avoids the interaction between too many variables, making it more widely applicable. As for the inclusion of multifocality, our model and the MSKCC, MDACC, and MOU models all indicate that multiple tumor foci impact non-SLN metastasis [27], although other investigators hold the opposite view [28]. This difference may be due to multifocality often being associated with large tumor volumes [29]. In our training group, the average tumor size of patients with multifocality was 3.64 cm (18/228). Moreover, a low occurrence rate of multiple foci may lead to distribution deviation. Differences in tumor cell aggressiveness, selection criteria, and sample size may also lead to different results.

Most models do not include the negative SLN number as a variable, the exceptions being the MSKCC and SCH models [22, 27]. Our model confirms that the negative SLN number significantly affects non-SLN metastasis. It is worth mentioning that the incidence of SLN metastasis is frequently accepted as an independent predictor, and both the Cambridge and Tenon models used this factor [26]. However, other studies tend to use the numbers for total SLNs, positive SLNs, and negative SLNs for analysis. The SLN metastasis rate actually combines the effect of positive SLNs and total SLNs, but the number of total SLNs does not necessarily affect the non-SLN state, as our results show. The SLN metastasis rate may also decrease the influence of the number of positive SLNs on the model. Therefore, we did not include it in our model.

Prior models rarely included age, menstrual state, and tumor location [14, 23, 30]. Our study yielded similar results. Breast cancer usually occurs in the breast's outer upper quadrant and rarely in the lower inner quadrant [31]. Although the outer upper quadrant is closer to the axilla, we did not find any correlation between tumor location and non-SLN metastasis. Metastasis development may depend more on the tumor's proliferative and invasive properties and the patient's internal environment. The classical MSKCC model includes the pathological subtype, which Mittendorf et al. [32] also regarded as an independent predictor. Some studies indicate that the pathology subtype does not affect non-SLN metastasis development [13, 25]. The failure to identify pathology subtype as an independent predictor in our study may be related to the different classifications of pathology subtypes. Insufficient sample size and uneven data distribution also make it challenging to reflect the pathology subtype's influence on non-SLN metastasis.

Histological grading, which involves assessing cancer cell division and differentiation, is an important indicator of cancer cell behavior. Although many investigators think that histological grading and lymph node metastasis may be related, the Cambridge model, which involved histological grading, had a multivariate analysis *P* value of 0.050 [12]. Degnim et al. conducted a meta-analysis of 11 studies. Among them, only one suggested that histological grading was correlated with non-SLN metastasis, while the others showed no correlation [33]. This study also supports the absence of correlation between histological grading and non-SLN metastasis. The higher the histological grading, the higher the degree of nuclear division, nuclear atypia, and vascularization. However, histological grading is often positively correlated with tumor size and vascular infiltration, so it is not necessarily an independent risk factor for non-SLN metastasis in statistical analyses. ER, PR, HER-2, and KI-67 are immunohistochemical indexes commonly associated with breast cancer and are closely related to patient treatment and prognosis. Most current models show no apparent correlation between KI-67 and the non-SLN status [13]. Whether the status of ER, PR, and HER-2 affects lymph node metastasis is still debated. ER was included in the nine variables in the MSKCC model, which analyzed the data of 11,946 patients and suggested that PR receptor status is related to axillary lymph node involvement [34]. Sandoughdaran et al. found HER-2 overexpression is related to non-SLN metastasis [35]. Mittendorf et al. and Fujii et al. pointed out that the ER/PR state and HER-2 expression are not significantly correlated with lymph node metastasis [32, 36]. Few models include molecular typing in their variables [37].

Most current models have an AUC between 0.60 and 0.78. With 228 patients in the training group and 60 patients in the validation group, our model yielded AUC values higher than 0.7 for both groups, indicating good performance [38]. The ASCO guidelines published in 2005 pointed out that SLNB has an average false-negative rate of about 8.4% (0%–29%) [7]. Therefore, we reasoned that a false-negative rate lower than 10% for axillary intervention would be acceptable to most physicians. Using the MSKCC and SCH models to explore low-risk interval [22], we found that, for risk values ≤10%, their respective false-negative rates were 4.86% and 3.54%. Furthermore, the corresponding proportion of patients was 8.10% and 13.6%, respectively. For risk values ≤15%, their respective false-negative rates were 13.54% and 8.20%, and the corresponding proportion of patients was 16.2% and 30.0%, respectively. In our model, for risk values ≤22.3%, the false-negative rate in the training group was only 1.8%, and the corresponding proportion of patients accounted for 4% of the total. For risk values ≤31.2%, the model could accurately identify about 13% of patients without non-SLN metastasis, while the false-negative rate was also below 10%.

Our model's low-risk interval value is higher than that of other models, but the proportion of patients avoiding ALND is lower. This phenomenon may be due to differences in the number and type of factors included in each model. In our model, the influence coefficients of multifocality and lymphovascular invasion are high, so the presence or absence of these two factors significantly impacts the prediction results. Furthermore, the training group contained only around 200 cases, making it hard to avoid the influence of risk interval division. However, our model can identify some non-SLN-negative patients with high accuracy as long as we strictly choose a low-risk cutoff value.

Axillary management of breast cancer has changed dramatically in the last decade. The IBCSG 23-01 trial in 2013 showed that if only SLN micrometastasis (<2 mm) occurs, omitting ALND does not negatively affect postoperative survival in early breast cancer patients [20]. In the EORTC-AMAROS trial, SLN-positive early patients were divided into a radiotherapy group and an ALND group. The two groups had similar 5-year recurrence rates (1.19% *vs.* 0.43%), but the radiotherapy group patients had a significantly higher quality of life than the ALND group patients [39]. The ACOSOG Z0011 trial in 2016 showed that postoperative radiotherapy for patients who accepted breast-conserving surgery can replace ALND, even if SLNB detects one or two positive SLNs, with no significant difference in overall survival, disease-free survival, or local recurrence between the groups [21]. However, those trials had relatively strict inclusion conditions and their results do not apply to most patients. The development of additional models can help more patients appropriately avoid ALND. Our model, which incorporates five common variables, demonstrates good prognostic performance in assessing non-SLN metastatic risk in positive SLN patients. However, it requires more external validation in the future.

## 5. Conclusion

Our study developed a new prognostic model capable of predicting the nonsentinel lymph node (non-SLN) status of Chinese breast cancer patients. The equation for predicting non-SLN metastasis includes the following factors: tumor size, multifocality, lymphovascular invasion, number of SLNs with metastasis, and number of SLNs without metastasis. Our model demonstrates good prognostic performance in assessing the risk of non-SLN metastasis in patients with metastasis-positive SLNs.

## Figures and Tables

**Table 1:** Comparison of clinicopathological characteristics between the model group and the validation group.

Data	Model group	Validation group	*P* value
Age			
≤35	16	2	0.382
>35	212	58	
Menopausal status			
Premenopausal	116	32	0.735
Postmenopausal	112	28	
Tumor location			
Upper outer	116	34	0.951
Lower outer	32	7	
Lower inner	13	3	
Upper inner	43	11	
Central	23	5	
Tumor size (cm)			
Mean	3.31	3.16	0.318
Median	3	3	
SD	1.29	0.95	
Tumor type			
Infiltrating ductal carcinoma	202	58	0.84
Invasive lobular carcinoma	9	0	
Other carcinomas	17	2	
Lymphovascular invasion			
Yes	31	2	0.023
No	197	58	
Histological grade			
G1	12	6	0.543
G2	69	19	
G3	136	33	
Gx	11	2	
Multifocality			
Yes	18	1	0.139
No	210	59	
Estrogen receptor			
Negative	59	15	0.890
Positive	169	45	
Progesterone receptor			
Negative	87	18	0.243
Positive	141	42	
HER2/neu receptor			
Negative	165	45	0.643
Positive	63	15	
Ki-67 status			
≤14%	33	12	0.294
>14%	195	48	
Molecular subtypes			
Luminal A	28	13	0.159
Luminal B1	113	20	
Luminal B2	29	8	
Her2-positive	34	10	
Triple negativity	24	9	
Number of SLN			
Mean	2.90	3.42	0.016
Median	3	3	
SD	1.48	1.44	
Number of metastatic SLN			
Mean	1.43	1.87	0.007
Median	1	1	
SD	0.73	1.16	
Number of nonmetastatic SLN			
Mean	1.46	1.55	0.663
Median	1	2	
SD	1.35	1.32	
Number of non-SLN			
Mean	13.05	14.37	0.264
Median	13	13	
SD	5.31	5.63	

**Table 2:** Univariate analysis of non-SLN status in 228 SLN-positive patients with early breast cancer.

Data	Non-SLN metastasis; absent; *n* = 116 (50.9%)	Non-SLN metastasis; present; *n* = 112 (49.1%)	Total *n* = 228	*P* value
Age				
≤35	12 (10.3%)	4 (3.6%)	16	0.045
>35	104 (89.6%)	108 (96.4%)	212	
Menopausal status				
Premenopausal	57 (49.1%)	59 (52.7%)	116	0.593
Postmenopausal	59 (50.9%)	53 (47.3%)	112	
Tumor location				
Upper outer	57 (49.6%)	59 (52.7%)	114	0.828
Lower outer	15 (13.0%)	17 (15.2%)	32	
Lower inner	6 (5.2%)	7 (6.2%)	13	
Upper inner	23 (20.0%)	20 (17.9%)	43	
Central	14 (12.2%)	9 (8.0%)	23	
Tumor size (cm)				
Mean	3.03	3.59	3.31	0.828
Median	3.00	3.40	3.00	
SD	1.11	1.39	1.29	
Tumor type				
Infiltrating ductal carcinoma	105 (90.5%)	97 (86.6%)	202	0.214
Invasive lobular carcinoma	2 (1.7%)	7 (6.3%)	9	
Other carcinomas	9 (7.8%)	8 (7.1%)	17	
Lymphovascular invasion				
Yes	8 (6.9%)	23 (20.5%)	31	0.003
No	108 (93.1%)	89 (79.5%)	197	
Histological grade				
G1	7 (6.0%)	5 (4.5%)	12	0.841
G2	37 (31.9%)	32 (28.5%)	69	
G3	66 (56.9%)	70 (62.5%)	136	
Gx	6 (5.2%)	5 (4.5%)	11	
Multifocality				
Yes	4 (3.4%)	14 (12.5%)	18	0.011
No	112 (96.6%)	98 (87.5%)	210	
Estrogen receptor				
Negative	34 (29.3%)	25 (22.3%)	59	0.228
Positive	82 (70.7%)	87 (77.7%)	169	
Progesterone receptor				
Negative	44 (37.9%)	43 (38.4%)	87	0.943
Positive	72 (62.1%)	69 (61.6%)	141	
HER2/neu receptor				
Negative	82 (70.7%)	83 (74.1%)	165	0.564
Positive	34 (29.3%)	29 (25.9%)	63	
Ki-67 status				
≤14%	19 (16.4%)	14 (12.5%)	33	0.405
>14%	97 (83.6%)	98 (87.5%)	195	
Molecular subtypes				
Luminal A	16 (13.8%)	12 (10.7%)	28	0.415
Luminal B1	51 (44.0%)	62 (55.4%)	113	
Luminal B2	17 (14.7%)	12 (10.7%)	29	
Her2-positive	17 (14.7%)	17 (15.2%)	34	
Triple negativity	15 (12.9%)	9 (8.0%)	24	
Number of SLN				
Mean	2.96	2.84	2.9	0.558
Median	3.0	3.0	3.0	
SD	1.53	1.43	1.48	
Number of metastatic SLN				
Mean	1.31	1.56	1.43	0.009
Median	1	1	1	
SD	0.58	0.84	0.73	
Number of nonmetastatic SLN				
Mean	1.65	1.28	1.46	0.034
Median	1	1	1	
SD	1.41	1.26	1.35	

**Table 3:** Multivariate analysis of non-SLN status in 228 SLN-positive patients with early breast cancer.

Factors	Coefficient	S.E.	Wald	*P*	OR	95% CI
Age						
≤35	1.000				1.000	
>35	0.990	0.633	2.447	0.118	2.691	0.779–9.302
Tumor size (cm)	0.267	0.123	4.734	0.030	1.307	1.027–1.663
Multifocality						
No	1.000				1.000	
Yes	1.443	0.599	5.801	0.016	4.235	1.308–13.709
Lymphovascular invasion						
No	1.000				1.000	
Yes	1.078	0.451	5.707	0.017	2.940	1.241–7.121
Number of metastatic SLN	0.471	0.221	4.552	0.033	1.602	1.039–2.469
Number of nonmetastatic SLN	−0.618	0.242	6.519	0.011	0.539	0.336–0.866
Constant	−2.541	0.802	10.030	0.002	0.079	

**Table 4:** The assignment table of independent factors about non-SLN metastasis.

Factors	Assignment
Tumor size = *a*	*a* = *x*_1,_*x*_1_ (cm) is the actual measured value in clinic
Multifocality = *b*	Yes = 1; No = 0
Lymphovascular invasion = *c*	Yes = 1; No = 0
Number of metastasis SLN = *d*	*d* = *x*_2_, *x*_2_ is the number of metastasis SLN
Number of nonmetastasis SLN = *e*	*e* = *x*_3_, *x*_3_ is the number of nonmetastasis SLN

**Table 5:** Diagnostic evaluation table about risk interval from the predictive equation in the model group.

Cutoff values	Non-SLN metastasis; absent	Non-SLN metastasis; present	Sensitivity	Specificity	False-negative rate (%)
≤14.2%	4 (2%)	0	100.0%	3.4%	0.0%
≤22.3%	9 (4%)	2	98.2%	6.8%	1.8%
≤28.6%	17 (7%)	6	94.6%	14.7%	5.4%
≤31.2%	30 (13%)	11	90.2%	22.4%	9.8%
≤35.7%	39 (17%)	17	84.8%	33.6%	15.2%
*n* = 228; among them, 112 patients were positive for non-SLNs with metastases					

**Table 6:** Diagnostic evaluation table about risk interval from the predictive equation in the validation group.

Cutoff values	Non-SLN metastasis; absent	Non-SLN metastasis; present	Sensitivity	Specificity	False-negative rate (%)
≤14.2%	0 (0%)	0	100.0%	0%	0.0%
≤22.3%	10 (17%)	3	90.3%	34.5%	9.7%
≤28.6%	16 (27%)	6	80.6%	55.2%	19.4%
≤31.2%	17 (28%)	8	77.4%	58.6%	22.6%
≤35.7%	18 (30%)	9	71.0%	58.6%	29%
*n* = 60; among them, 31 patients were positive for non-SLN metastases					

## Data Availability

All data of this study are available from the corresponding author upon request.
